# Stevens–Johnson syndrome/toxic epidermal necrolysis and erythema multiforme drug-related hospitalisations in a national administrative database

**DOI:** 10.1186/s13601-017-0188-1

**Published:** 2018-01-22

**Authors:** Bernardo Sousa-Pinto, Luís Araújo, Alberto Freitas, Osvaldo Correia, Luís Delgado

**Affiliations:** 1 Basic and Clinical Immunology, Department of Pathology, Faculty of Medicine, University of Porto, Alameda Prof. Hernâni Monteiro, 4200-319 Porto, Portugal; 2 MEDCIDS - Department of Community Medicine, Information and Health Decision Sciences, Faculty of Medicine, University of Porto, Rua Dr. Placido da Costa, 4200-450 Porto, Portugal; 3 CINTESIS – Center for Health Technology and Services Research, Rua Dr. Placido da Costa, 4200-450 Porto, Portugal; 4 Allergy Unit, CUF Institute, Porto, Portugal; 5 Epidermis Dermatology Center, CUF Institute, Porto, Portugal

**Keywords:** Epidemiology, Erythema multiforme, Drug allergy, Stevens–Johnson syndrome, Toxic epidermal necrolysis

## Abstract

**Background:**

Stevens–Johnson syndrome/toxic epidermal necrolysis (SJS/TEN) and erythema multiforme (EM) are immunologically-mediated dermatological disorders commonly triggered by drug exposure and/or other external agents. We aimed to characterise SJS/TEN- and EM-drug-related hospitalisations in a nationwide administrative database, focusing on demographic and clinical characteristics, and in the most frequently implicated drug classes.

**Methods:**

We analysed all drug-related hospitalisations with associated diagnosis of SJS/TEN or EM in Portuguese hospitals between 2009 and 2014. We compared gender, age, comorbidities, length of stay, and in-hospital mortality and estimated the number of episodes per million packages sold of drug classes. Predictors of in-hospital mortality were investigated in both conditions by logistic regression.

**Results:**

There were 132 SJS/TEN-related and 122 EM-related hospitalisations. Incidence and in-hospital mortality of SJS/TEN episodes (24.2%) were consistent with previous studies. HIV co-infection was more common among SJS/TEN hospitalisations (9 vs. 2% with EM; *P* = 0.009). Liver disease, advanced age, and a TEN diagnosis, were significantly associated with higher risk of mortality in patients with SJS/TEN. The highest numbers of SJS/TEN and EM episodes per million drug packages sold were observed for antivirals (8.7 and 1.5, respectively), antineoplastic/immunosuppressive drugs (5.6 and 3.9, respectively) and hypouricaemic drugs (5.0 and 2.4, respectively).

**Conclusions:**

SJS/TEN in-hospital mortality is high, and its risk factors include advanced age, liver disease, and TEN diagnosis. The drug classes most frequently associated with these conditions include antivirals, hypouricaemic drugs and antineoplastic/immunosuppressive drugs. Administrative databases seem useful in the study of SJS/TEN drug-related hospitalisations, yielding results consistent with previous studies and on a nationwide basis.

**Electronic supplementary material:**

The online version of this article (10.1186/s13601-017-0188-1) contains supplementary material, which is available to authorized users.

## Background

Severe cutaneous adverse reactions (SCARs) are an example of severe type B adverse drug reactions, and are associated with high morbidity and mortality [1]. SCARs encompass three distinct clinical entities: (1) the spectrum of Stevens–Johnson syndrome/toxic epidermal necrolysis (SJS/TEN), (2) acute generalised exanthematous pustulosis (AGEP), and (3) drug reactions with eosinophilia and systemic symptoms (DRESS) [2, 3]. Diagnosis of these conditions is further complicated by the existence of overlap syndromes, characterised by the coexistence of features from different entities [4]. The SJS/TEN spectrum is the most common and lethal of all SCARs. It associates with a mortality of up to 40%, versus just under 5% for AGEP and 10% for DRESS [5, 6]. SJS/TEN is characterised by cutaneous detachment and blister formation—in SJS, skin detachment affects less than 10% of the body surface area, while TEN requires involvement of over 30% [7, 8]. Cases with 10–30% of body surface area involvement are classified as SJS–TEN overlap syndrome [7, 8].

Until recently, there was widespread belief that erythema multiforme (EM) major was a milder form of SJS/TEN spectrum [9, 10]. However, this assumption has now been largely abandoned—most cases of EM are associated with herpes virus infections, while only a minority are deemed to be caused by drugs [9–12]. Distinction between EM and SJS/TEN is crucial since the latter is associated with much greater severity and higher mortality [9], and as these conditions have different treatment approaches. Clinically, SJS/TEN is characterised by macules or flat atypical target lesions with widespread distribution or preferential trunk involvement, which rapidly evolve as a blistering disorder of the skin and mucosal surfaces. Conversely, EM typically presents with predominantly acral target lesions [11, 13]. Nevertheless, atypical presentations can make it difficult to distinguish between the two entities [13], especially at the beginning of the clinical presentation and when there is a history of previous drug exposure.

An improved knowledge on the risk factors associated with SJS/TEN might thus facilitate the distinction between SJS/TEN and EM, as well as provide clues concerning the pathophysiology of this condition [11, 14, 15]. However, is spite of its severity, the epidemiology of SJS/TEN remains insufficiently studied [16], in part because its rarity renders traditional case–control or cohort studies particularly time- and resource-consuming. On the other hand, administrative databases are being increasingly used in the assessment of such rare and very rare conditions [16]. Therefore, in this study, we analysed a nationwide administrative database with the aim of characterising drug-related hospitalisations in patients with SJS/TEN, with a focus on gender and age, comorbidities, length of hospital stay, in-hospital mortality, and responsible drug classes. We compared these results to those observed for patients with a diagnosis of drug-related EM, so that we could infer whether in administrative databases SJS/TEN cases are mostly distinguished from other often confused conditions.

## Methods

We used a database provided by the Portuguese Central Health System Administration containing data for all hospitalisations in mainland Portugal public hospitals. Anonymity was maintained for all hospitals and patients. For each episode, we had access to the main diagnosis (clinical condition responsible for the patient’s admission), up to 19 accompanying diagnoses, and up to 5 external causes of injury and poisoning (including adverse drug effects). Both diagnoses and external causes had been coded with ICD-9-CM codes after discharge; thus, both community cases requiring hospitalisation and in-hospital cases were identified. Coding in Portugal is standardised and performed by doctors with specific training, and internal and external auditing is regularly performed to ensure proper coding [17].

We analysed all hospitalisations with a main or supplementary diagnosis of SJS/TEN (ICD-9-CM codes 695.13–695.15) and an associated E code (ICD-9-CM codes E930.x–E949.x for adverse drug reactions—ICD-9-CM codes are listed in Additional file 1: Table S1—each code corresponds to the drug class deemed responsible for the reaction according to the responsible physician). We separately analysed hospitalisations with an associated diagnosis of SJS (695.13), SJS–TEN overlap (695.14), and TEN (695.15) to allow for comparison between these conditions. Since these three codes were introduced in October 2008 [18], we only analysed hospitalisations between January 2009 and December 2014. SJS/TEN episodes were compared with hospitalisations with main or supplementary diagnosis of EM (ICD-9-CM codes 695.10, 695.11, 695.12, and 695.19) and an associated E code.

We calculated the number of hospitalisations of SJS/TEN and EM per million inhabitants based on data published by the Portuguese National Institute of Statistics [19]. This rate probably provides a good estimation of the 6-year incidence of SJS/TEN in Portugal as the severity of this condition entails almost all patients to be hospitalised in public hospitals.

We compared gender, age, and comorbidities between episodes with a diagnosis of EM and hospitalisations with a diagnosis of SJS/TEN; the three clinical entities of the latter (SJS, SJS–TEN overlap, and TEN) were also compared with each other. We compared the frequency of comorbidities potentially associated with an increased risk of SJS/TEN (whether directly or indirectly), namely chronic kidney disease, hypertension, heart failure, diabetes, HIV infection, and liver disease (Additional file 1: Table S1). Chronic kidney disease is associated with an increased risk of allopurinol-induced SJS/TEN [20]. As the use of diuretics is also associated with the latter condition [21], we also assessed conditions whose treatment frequently requires the use of diuretics, namely hypertension, heart failure, and diabetes. HIV infection was assessed, as the risk of SJS/TEN is known to be higher among HIV^+^ patients [22]. Liver disease may also be a risk factor for SJS/TEN, particularly in regard to chronic viral hepatitis [23]. We also evaluated length of hospital stay, readmission rate, and in-hospital mortality. To study hospital readmissions, we identified individual patients admitted between 2009 and 2014 and followed them up until the end of the study period. Registries in our database had been anonymised and, therefore, individual patients were identified according to their gender, birthdate and residence—two episodes were deemed to have occurred with the same patient whenever the registered inpatient’s gender, birthdate and residence were equal, and the registered diagnoses were similar.

For each clinical entity, we compared the frequency of the drug classes recorded as implicated in the adverse drug reactions. In the dataset used, adverse drug reactions are identified by E codes, each of which corresponds to a different class of drugs. In addition, based on information provided by the Portuguese Authority of Medicines and Health Products (INFARMED), we estimated the number of EM and SJS/TEN episodes per million packages of drugs sold [24–28]. For this estimate, we excluded data from 2009 due to the risk of underreporting, as the ICD-9-CM SJS/TEN codes were introduced that year.

Categorical variables were compared using the Chi square test and the Fisher exact test. Continuous variables were analysed using the Mann–Whitney U test and the Kruskal–Wallis test. *P* values < 0.05 were considered statistically significant. To analyse risk factors significantly associated with in-hospital mortality (both for EM and SJS/TEN), we used logistic regression models. We performed univariable analyses assessing the association between in-hospital mortality and gender, age, length of hospital stay, SJS/TEN entities (SJS, SJS–TEN overlap, and TEN), hypertension, diabetes, heart failure, chronic kidney failure, liver disease, and HIV status. Variables with marginal association in the univariate analysis (*P* < 0.20) were included in multivariable models. The models were assessed by their area under the receiver operating characteristics curve (AUC-ROC) and by the Hosmer–Lemeshow goodness-of-fit test; multicollinearity was assessed using variance inflation factor. The results of the univariable and multivariable analyses are expressed as odds ratio (OR) with 95% confidence intervals (95% CI), and *P* values. All statistical analyses were performed using SPSS version 22.0 (IBM^®^SPSS^®^ Statistics, Armonk, NY:IBM Corp.).

For this study, Ethics Committee Approval and Informed consent were not needed, as all data had previously been anonymised.

## Results

From 2009 to 2014, we recorded 122 hospitalisations with an associated diagnosis of EM (main diagnosis in 34 cases) and 132 hospitalisations with an associated diagnosis of SJS/TEN (main diagnosis in 89 cases). This corresponds to a 6-year incidence of 13.2 hospitalisations with EM and 12.2 hospitalisations with SJS/TEN per million inhabitants (Table 1). In 2014, we observed a 1-year incidence of 2.1 EM hospitalisations and 3.8 SJS/TEN hospitalisations per million inhabitants, while the 1-year incidences in 2009 were of 3.2 EM hospitalisations and 0.7 SJS/TEN hospitalisations per million inhabitants (Additional file 2: Table S2), suggesting a learning effect as SJS/TEN codes were firstly used in 2009.

**Table 1:** Demographic and clinical characteristics of hospitalised patients with an associated diagnosis of erythema multiforme (EM) or Stevens–Johnson syndrome/toxic epidermal necrolysis (SJS/TEN); Mainland Portugal, 2009–2014 (*n* = 254 hospitalisations)

Characteristics	Cutaneous adverse reactions			SJS/TEN			
	EM^a^ (N = 122)	SJS/TEN (N = 132)	*P* value	Stevens–Johnson Syndrome (N = 73)	SJS–TEN overlap^b^ (N = 18)	Toxic epidermal necrolysis (N = 41)	*P* value
Episodes as main diagnosis—n (%)	34 (27.9)	89 (66.4)		45 (61.6)	14 (77.8)	30 (68.3)	
6-years incidence (*per* million inhabitants)	12.2	13.2		7.3	1.8	4.1	
Gender—n (%)							
Male	43 (35.2)	60 (45.5)	0.098	34 (46.6)	9 (50.0)	17 (41.5)	0.798
Female	79 (64.8)	72 (54.5)		39 (53.4)	9 (50.0)	25 (58.5)	
Age (years)							
Median (percentile 25–75)	63 (44–77)	63 (45–75)	0.903	64 (46–79)	57 (34–72)	65 (48–74)	0.492
Comorbidities							
Hypertension	39 (32.0)	46 (34.8)	0.627	29 (39.7)	5 (27.8)	12 (29.3)	0.422
Diabetes	19 (15.6)	22 (16.7)	0.813	13 (17.8)	4 (22.2)	5 (12.2)	0.589
Heart failure	10 (8.2)	13 (9.8)	0.647	8 (11.0)	3 (16.7)	2 (4.9)	0.272
Chronic kidney disease	12 (9.8)	15 (11.4)	0.693	7 (9.6)	4 (22.2)	4 (9.8)	0.340
Liver disease	11 (9.0)	10 (7.6)	0.677	4 (5.5)	3 (16.7)	3 (7.3)	0.203
Acute toxic hepatitis	7 (5.7)	6 (4.5)	0.882	2 (2.7)	2 (11.1)	2 (4.9)	0.269
HIV	2 (1.6)	12 (9.1)	0.009	8 (11.0)	3 (16.7)	1 (2.4)	0.115
Length-of-stay^c^ (days)							
Median (percentile 25–75)	10 (5–20)	15 (7–28)	0.007	15 (7–23)	23 (9–36)	14 (7–28)	0.456
In-hospital mortality—n (%)	9 (7.4)	32 (24.2)	< 0.001	12 (16.4)	2 (11.1)	18 (43.9)	0.002

^a^Encompasses 5 cases of EM minor, 11 cases of EM major, 10 cases of other forms of EM, and 96 cases of unspecified EM

^b^Stevens–Johnson and toxic epidermal necrolysis overlap syndrome

^c^Includes all episodes, including those resulting in the death of patients

SJS (*n* = 73) accounted for 55% of all SJS/TEN hospitalisations, while SJS–TEN overlap (*n* = 18) and TEN (*n* = 41) accounted for 14% and 31%, respectively (Table 1). Patient readmissions accounted for 3% (*n* = 7) of all hospitalisations (2% for EM vs. 4% for SJS/TEN). All readmissions occurred within 1 year of the first hospitalisation and most cases (all EM readmissions and 33% of SJS/TEN readmissions) were due to exposure to the same drug class. A majority of hospitalisations occurred in females, both for EM (65%) and SJS/TEN (55%). The median age in each case was 63 years. Six percent (*n* = 7) of EM episodes and 8% (*n* = 10) of SJS/TEN episodes (8 with SJS and 2 with TEN diagnosis) occurred in children, and 43% and 70% occurred, respectively, in girls. No significant differences were observed for gender or age distribution between the distinct SJS/TEN entities (SJS, SJS–TEN overlap, and TEN).

HIV co-infection was more common in hospitalisations with associated diagnosis of SJS/TEN (9%) than with EM (2%) (*P* = 0.009). No significant differences were observed for the frequency of any of the other comorbidities studied.

The median length of hospital stay was 10 days for EM versus 15 days for SJS/TEN (*P* = 0.007). Within the SJS/TEN group, TEN episodes were associated with the shortest median length of stay (14 days); however, considering non-fatal cases only, TEN was associated with a median length of stay of 22 days.

A fatal outcome was reported for 7% of hospitalisations with associated diagnosis of EM and for 24% of SJS/TEN episodes (*P* < 0.001). In the SJS/TEN group, TEN had the highest proportion of fatal cases (44%), followed by SJS (16%), and SJS–TEN overlap (11%) (*P* = 0.002). No fatal cases were registered among paediatric patients. Variables significantly associated with in-hospital mortality in hospitalisations with a diagnosis of SJS/TEN after multivariable analysis included advanced age (OR 1.1 per year; 95% CI 1.0–1.1; *P* = 0.002), a diagnosis of liver disease (OR 7.9; 95% CI 1.2–50.7; *P* = 0.031), and TEN (OR 6.5; 95% CI 2.3–18.8; *P* = 0.001) (Table 2). For EM, the variables significantly associated with in-hospital mortality were advanced age (OR 1.1 per year; 95% CI 1.0–1.2; *P* = 0.005) and male gender (OR 23.9; 95% CI 3.4–168.6; *P* = 0.001). The multivariable models for SJS/TEN and EM hospitalisations had an AUC-ROC of 0.843 and 0.912, respectively. The Hosmer–Lemeshow goodness-of-fit test did not evidence lack of fit in the multivariable model for SJS/TEN (*P* = 0.942) or EM (*P* = 0.995). The models did not show evidence of multicollinearity.

**Table 2:** Results from binomial logistic regression with in-hospital mortality as the dependent variable

	Erythema multiforme		Stevens–Johnson syndrome/toxic epidermal necrolysis	
	Crude OR (95% CI); *P* value	Adjusted OR (95% CI); *P* value^a^	Crude OR (95% CI); *P* value	Adjusted OR (95% CI); *P* value^b^
Male gender	7.5 (1.5–37.9); 0.015	23.9 (3.4–168.6); 0.001	1.4 (0.6–3.1); 0.432	–
Age	1.1^c^ (1.0–1.2); 0.011	1.1^c^ (1.0–1.2); 0.005	1.1^c^ (1.0–1.1); < 0.001	1.1^c^ (1.0–1.1); 0.002
Length of stay	1.0^d^ (0.9–1.1); 0.750	–	1.0^d^ (1.0–1.0); 0.398	–
Hypertension	2.9 (0.7–11.5); 0.129	0.7 (0.1–4.4); 0.721	0.9 (0.4–2.0); 0.729	–
Diabetes	1.6 (0.3–8.4); 0.571	–	1.7 (0.6–4.6); 0.316	–
Heart failure	1.4 (0.2–12.9); 0.742	–	4.6 (1.4–15.0); 0.011	4.1 (0.9–18.2); 0.060
Chronic kidney disease	2.9 (0.5–16.1); 0.213	–	2.5 (0.8–7.6); 0.117	0.7 (0.2–3.1); 0.725
Liver disease	^e^	^e^	3.7 (1.0–13.7); 0.051	7.9 (1.2–50.7); 0.031
HIV	^e^	^e^	0.6 (0.1–3.0); 0.562	–
Clinical entity				
Stevens–Johnson syndrome	–	–	1.0^f^	1.0^f^
Stevens–Johnson syndrome–toxic epidermal necrolysis overlap	–	–	0.6 (0.1–3.1); 0.577	0.4 (0.1–3.4); 0.437
Toxic epidermal necrolysis	–	–	4.0 (1.7–9.5); 0.002	6.5 (2.3–18.8); 0.001

*OR* odds ratio, *CI* confidence interval

^a^Adjusted for gender, age, and hypertension

^b^Adjusted for age, Stevens–Johnson syndrome, toxic epidermal necrolysis, heart failure, chronic kidney disease, and liver disease

^c^Values per year

^d^Values per day

^e^No fatal cases were registered

^f^Reference category

Drug classes most frequently associated with adverse reactions in hospitalised patients with SJS/TEN were antibiotics (26%), uric acid metabolism drugs (20%), and anticonvulsants (17%) (Table 3). The same drug classes were identified in cases of EM: 30% for antibiotics, 17% for uric acid metabolism drugs, and 11% for anticonvulsants. In paediatric patients, antibiotics were responsible for the greatest proportion of adverse reactions in EM (57%) and SJS/TEN (30%) hospitalisations. The two registered cases of TEN in children were associated with antiviral and psychotropic drugs.

**Table 3:** Drug classes deemed responsible for the cutaneous adverse reactions occurred in the context of hospitalisations with an associated diagnosis of erythema multiforme (EM) or Stevens–Johnson syndrome/toxic epidermal necrolysis (SJS/TEN); Mainland Portugal, 2009–2014 (*n* = 254 hospitalisations)

Drug class—n (%)	Cutaneous adverse reactions			SJS/TEN			
	EM (N = 122)	SJS/TEN (N = 132)	*P* value	SJS (N = 73)	SJS–TEN overlap^a^ (N = 18)	TEN (N = 41)	*P* value
Antibiotics	36 (29.5)	34 (25.8)	0.504	18 (24.7)	6 (33.3)	10 (24.4)	0.731
Penicillins	9 (7.4)	9 (6.8)	0.862	2 (2.7)	4 (22.2)	3 (7.3)	0.016
Other specified antibiotics^a^	22 (18.0)	22 (16.7)	0.774	14 (19.2)	1 (5.6)	7 (17.1)	0.380
Unspecified antibiotics	4 (3.3)	2 (1.5)	0.431	1 (1.4)	–	1 (2.4)	0.999
Other anti-infectives	10 (8.2)	18 (13.6)	0.167	12 (16.4)	4 (22.2)	2 (4.9)	0.117
Sulfonamides	4 (3.3)	8 (6.1)	0.296	5 (6.8)	2 (11.1)	1 (2.4)	0.285
Antimycobacterial drugs	1 (0.8)	2 (1.5)	0.999	2 (2.7)	–	–	0.654
Antiviral drugs	1 (0.8)	7 (5.3)	0.068	4 (5.5)	2 (11.1)	1 (2.4)	0.340
Hormones and synthetic substitutes	7 (5.7)	7 (5.3)	0.879	5 (6.8)	2 (11.1)	–	0.126
Antineoplastic and immunosuppressive drugs^b^	13 (10.7)	7 (5.3)	0.114	3 (4.1)	1 (5.6)	3 (7.3)	0.759
Agents primarily affecting blood constituents	–	4 (3.0)	0.123	2(2.7)	1 (5.6)	1 (2.4)	0.611
Analgesics, antipyretics, and antirheumatics	11 (9.0)	7 (5.3)	0.249	3 (4.1)	1 (5.6)	3 (7.3)	0.759
Anticonvulsants	13 (10.7)	22 (16.7)	0.165	12 (16.4)	2 (11.1)	8 (19.5)	0.726
Sedatives and hypnotics	–	1 (0.8)	0.999	–	1 (5.6)	–	0.136
Psychotropic agents	2 (1.6)	4 (3.0)	0.685	2 (2.7)	–	2 (4.9)	0.789
Agents primarily affecting the cardiovascular system	2 (1.6)	2 (1.5)	0.999	1 (1.4)	–	1 (2.4)	0.999
Uric acid metabolism drugs^c^	21 (17.2)	26 (19.7)	0.611	17 (23.3)	2 (11.1)	7 (17.1)	0.447
Agents primarily acting on the smooth and skeletal muscles and respiratory system	1 (0.8)	–	0.480	–	–	–	
Agents primarily affecting skin and mucous membrane, ophthalmological, otorhinolaryngological and dental drugs	1 (0.8)	–	0.480	–	–	–	
Other and unspecified drugs and medicinal substances^d^	9 (7.4)	12 (9.1)	0.620	7 (9.6)	–	5 (12.2)	0.368

^a^Includes, among others, macrolides, tetracyclines, and cephalosporins

^b^These were the only drugs of the class “primarily systemic agents” in which cutaneous adverse reactions were registered

^c^These were the only drugs of the class “water, mineral, and uric acid metabolism drugs” in which cutaneous adverse reactions were registered

^d^There were no cutaneous adverse reactions associated with use of drugs belonging to the classes “other central nervous system depressants and anesthetics”, “central nervous system stimulants”, “drugs primarily affecting the autonomic nervous system” and “agents primarily affecting gastrointestinal system”

Eighty-eight percent of episodes with reported adverse reactions to antivirals were in HIV^+^ patients. In hospitalisations with associated diagnosis of EM, chronic kidney disease was more common in episodes with adverse reactions attributed to uric acid metabolism drugs (*P* = 0.006). In hospitalisations with SJS/TEN, however, chronic kidney disease was significantly associated with adverse reactions to antibiotics (*P* = 0.023). Liver disease was not significantly associated with adverse reactions to any of the drug classes analysed.

The drug classes associated with a higher number of SJS/TEN episodes per million packages sold were antiviral drugs (8.7 episodes), followed by anti-neoplastic/immunosuppressive drugs (5.6), uric acid metabolism drugs (5.0), and anticonvulsants (1.2). The corresponding classes for EM were anti-neoplastic/immunosuppressive drugs (3.9 episodes), followed by uric acid metabolism drugs (2.4) and antiviral drugs (1.5)(Table 4).

**Table 4:** Number of hospitalisations with an associated diagnosis of erythema multiforme (EM) and Stevens–Johnson syndrome/toxic epidermal necrolysis (SJS/TEN) per million sold packages of drug classes involved in adverse reactions; Mainland Portugal, 2010–2014 (*n* = 215 hospitalisations)

Drug class	EM (N = 90)	SJS/TEN (N = 125)
Antibiotics (NPS = 37,311,945)	0.75	0.88
Antiviral drugs (NPS = 686,221)	1.46	8.74
Hormones and synthetic substitutes (NPS = 69,570,259)	0.08	0.10
Antineoplastic and immunosuppressive drugs (NPS = 1,787,045)	3.92	5.60
Agents primarily affecting blood constituents (NPS = 37,608,542)	–	0.11
Analgesics, antipyretics, and antirheumatics (NPS = 71,419,516)	0.12	0.10
Anticonvulsants (NPS = 16,305,856)	0.43	1.17
Psychotropic agents (NPS = 100,008,577)	0.01	0.03
Agents primarily affecting the cardiovascular system (NPS = 198,300,264)	0.01	0.01
Uric acid metabolism drugs (NPS = 5,007,028)	2.40	4.99

*NPS* number of drug packages sold

## Discussion

We used an administrative database to assess SJS/TEN and EM hospitalisations, and found that hospitalisations in patients with SJS/TEN were associated with significantly longer hospital stays and higher in-hospital mortality than hospitalisations in patients with associated diagnosis of EM, highlighting the need to accurately distinguish between these two clinical conditions. Drug classes responsible for the greatest proportion of adverse reactions in patients with SJS/TEN were antibiotics, uric acid metabolism drugs, and anticonvulsants. In-hospital mortality in SJS/TEN cases was significantly associated with liver disease, advanced age, and TEN diagnosis.

Most hospitalisations with associated diagnosis of SJS/TEN and EM occurred in females and older patients, a finding not totally consistent with several other studies, which found EM to be more frequent among males and younger patients [11, 29]. One the one hand, it is possible to hypothesise that this study predominantly assessed severe cases of EM, as most cases of this condition do not require hospitalisation (i.e. SJS/TEN episodes regardless of their severity were only compared with the most severe EM cases). However, it is also possible to infer that a substantial number of cases classified with a diagnosis of “EM” might actually consist of misclassified cases. In fact, the diagnosis of “drug-related EM” appears to be over-attributed [30]. A recent review found that, from 36 articles published from 2010 to 2016 and describing putative cases of drug-related EM, only 6 described cases compatible with probable/definite EM [30].

In our database analysis, HIV co-infection was significantly more common among SJS/TEN hospitalisations. Previous studies have found that HIV^+^ patients have a higher risk of developing SJS and TEN [22]. In fact, HIV^+^ patients are more likely to use some of the drugs most frequently implicated in SJS/TEN, such as antiretrovirals, sulfamethoxazole/trimethoprim, and antituberculosis drugs, and they often use them at higher doses [31, 32]. Secondly, these patients have a decreased number of skin CD_4_^+^ regulatory T cells and appear to have altered drug metabolism [31, 32]. HIV might also contribute to the pathogenesis and local cytotoxic mechanisms of SJS/TEN, as suggested by the presence of HIV antigens in the skin lesions of patients with these reactions [33].

The frequency of fatal cases in our series is consistent with other studies [34, 35]. Advanced age was identified as a risk factor for in-hospital mortality among SJS/TEN episodes; in fact, advanced age is an independent risk factor in the SCORTEN severity scale [36, 37]. Liver disease was also found to be associated with increased in-hospital mortality in our analysis, and while hepatic involvement in SCARs is associated with high mortality [38], chronic viral hepatitis has also been hypothesised by some authors to be a risk factor for TEN [23]. Impaired drug metabolism secondary to chronic liver disease could also enhance the risk of a fatal outcome.

The drug classes responsible for adverse reactions most frequently associated with SJS/TEN included antibiotics, antivirals, anticonvulsants, and uric acid metabolism drugs. Although we were not able to identify the specific culprit drugs within these groups, the latter include drugs that have frequently been previously described to be associated with SCARs, such as allopurinol, and lamotrigine [14, 15]. After adjusting for the number of drug packages sold, we also found a high rate of hospitalisations associated with adverse reactions to antineoplastic and immunosuppressive drugs, supporting some underlying immune deregulation associated with malignancies or autoimmune diseases [14]. While many cases of EM and SCARs have been reported following the use of several of these drugs, the underlying immunological mechanisms are still only partially identified [20, 39, 40]. Some antineoplastic drugs (e.g., EGFR tyrosine kinase inhibitors) interfere with keratinocyte proliferation, differentiation, and migration, and, thus, might facilitate the development of more severe SCARs. Similarly, radiotherapy could also increase the risk of EM and SCARs, possibly by inhibiting hepatic enzymes responsible for drug metabolism [41].

The use of an administrative database to study rare conditions such as SJS/TEN might have some advantages comparing to traditional case–control and cohort studies. Although these are the ideally preferred studies, case–control and cohort studies are usually resource-consuming and difficult to conduct, particularly on a nationwide and frequent basis [16, 42–44]. Additionally, administrative database studies may yield results consistent with these registry-based studies; for instance, the results described in our study concerning SJS/TEN demographic characteristics and mortality are similar with those of a recent study conducted in Italy [14]. On the other hand, in the lack of other registries, administrative database studies might feasibly complement pharmacovigilance studies and help to detect regional differences. The comprehensiveness of administrative databases regarding this condition is another important advantage—due to its severity, SJS/TEN is a condition requiring hospitalisation; additionally, the nationwide scope of the database allows for an overcome of possible biases related to the assessment of participants of a single region [16].

Nevertheless, although coding is standardised and frequently audited in Portugal, it should be noted that these databases might be incomplete or inaccurate [42]. A systematic review found that only 53–60% of ICD-9-CM code 695.1 reports consisted of validated cases of EM, SJS and TEN [45], while Davis et al. [18] found that, among inpatients, ICD-9-CM codes 695.13–695.15 correctly identified 50% of patients, and up to 57–92% when only patients hospitalised for three or more days were considered. While, in our study, we did not select patients according to their length of stay, only three patients had been hospitalised for less than 3 days (two of them died when hospitalised). Additionally, we identified drug hypersensitivity cases by using a combination of both ICD-9-CM diagnostic codes and E codes. According to Saff et al. [46], this combination identifies drug allergy patients more accurately than the use of a single code, but it underestimates the true incidence of drug allergic reactions. This algorithm lacks, however, validation regarding episodes with associated diagnosis of drug-related EM. In fact, not only there are several drug-related conditions which do not have a specific ICD-9-CM code (e.g. DRESS and AGEP), but also some heterogeneous drug-induced skin-eruptions may present as EM-like and, therefore, might be misclassified as EM [47, 48]. While these conditions do not have a specific ICD-10 code either [49], they are planned to have a specific ICD-11 code [50, 51]—in fact, with the development and adoption of ICD-11, the accuracy of administrative databases in the assessment of SCARs may improve, as a greater diversity of diagnosis procedures, drugs (and not only drug classes) and clinical entities have been ascribed specific codes—EJ00-EJ18 codes concern “adverse cutaneous reactions to medication” and include, among others, specific codes for DRESS, AGEP and fixed drug eruption). Additionally, it is paramount to ensure the validity of the hospital discharge codes for EM [18], as well as to educate clinicians on the differential diagnoses of drug-related skin disorders [30].

Another major limitation concerns the impossibility of identifying the specific drugs associated with each episode. Thus, it is only possible to speculate about the identity of the culprit drugs. For instance, it is highly probable that most hypersensitivity reactions to uric acid metabolism drugs were due to allopurinol, while most cutaneous reactions to antivirals were probably associated with antiretroviral drugs, since these reactions mostly occurred in HIV^+^ patients. We also lack information on the specific clinical presentation of each episode, criteria used by the physicians to deem a specific drug class responsible for the corresponding reactions, co-occurrence of herpes reactivation and patient ethnicity or birthplace. Another possible limitation concerns the indirect method used to identify hospital readmissions (based on inpatients’ gender, birth date and residence). Although this method does not identify distinct patients with complete certainty, we confirmed that episodes identified as readmissions and the respective “first admissions” had a similar set of associated diagnoses and, for the cases occurred in the same hospital, an equal hospital-specific inpatient identifier number.

## Conclusions

In this administrative database-analysis, SJS and TEN were associated with higher in-hospital mortality and longer hospital stays than other drug-related mucocutaneous conditions. In hospitalisations with a diagnosis of drug-related SJS or TEN, an increased risk of in-hospital mortality was associated with advanced age, with a TEN diagnosis, and liver disease. Our findings provide an epidemiological characterisation of SJS/TEN hospitalisations, as well as an identification of factors significantly associated with higher in-hospital mortality. This epidemiological knowledge might prove useful for performing an earlier diagnosis of SJS/TEN, allowing for an earlier start of the most adequate therapy; additionally, identifying factors associated with higher fatality will be essential for defining the most appropriate measures to prevent fatal outcomes. These results suggest that administrative databases are useful in the assessment of SJS/TEN drug-related hospitalisations in a nationwide basis, allowing for epidemiological studies to be conducted in a frequent- and low-resource-consuming basis. While this may be particularly advantageous for obtaining knowledge on SJS/TEN and for healthcare planning, further studies on this methodological approach are needed.

## Additional files

**Additional file 1: Table 1.** ICD-9-CM codes used to identify the assessed conditions and external causes of injury.

**Additional file 2: Table 2.** Annual incidence of hospitalisations with associated diagnosis of erythema multiforme (EM) or Stevens-Johnson syndrome/toxic epidermal necrolysis(SJS/TEN).

## Abbreviations

AGEP: acute generalised exanthematous pustulosis
AUC-ROC: area under the receiver operating characteristics curve
CI: confidence interval
DRESS: drug reactions with eosinophilia and systemic symptoms
EM: erythema multiforme
OR: odds ratio
SCARs: severe cutaneous adverse reactions
SJS: Stevens–Johnson syndrome
TEN: toxic epidermal necrolysis
