# Supplementary material for: Stevens–Johnson syndrome/toxic epidermal necrolysis and erythema multiforme drug-related hospitalisations in a national administrative database
Source: Clin Transl Allergy. 2018 Jan 22;8:2. doi: 10.1186/s13601-017-0188-1 (PMC5776772; doi:10.1186/s13601-017-0188-1)
Supplement: Supplementary file 1 — Additional file 1: Table 1. ICD-9-CM codes used to identify the assessed conditions and external causes of injury. [file 13601_2017_188_MOESM1_ESM.docx]

**Additional table 1.** ICD-9-CM codes used to identify the assessed conditions and external causes of injury.

| A. ICD-9-CM diagnosis codes used to identify Stevens-Johnson syndrome/toxic epidermal necrolysis and erythema multiforme | | |
| --- | --- | --- |
| ICD-9-CM codes | **Description** | |
| 695.10 ^a^ | Erythema multiforme, unspecified |  |
| 695.11 ^a^ | Erythema multiforme minor |  |
| 695.12 ^a^ | Erythema multiforme major |  |
| 695.13 | Stevens-Johnson syndrome |  |
| 695.14 | Stevens-Johnson syndrome-toxic epidermal necrolysis overlap syndrome |  |
| 695.15 | Toxic epidermal necrolysis |  |
| 695.19 ^a^ | Other erythema multiforme |  |
| B. ICD-9-CM external cause codes of “drugs, medicinal and biological substances causing adverse effects in therapeutic use” | | |
| ICD-9-CM codes | **Description** | |
| E930.x | Antibiotics |  |
| E930.0 | Penicillins |  |
| E930.1-E930.8 | Other specified antibiotics ^b^ |  |
| E930.9 | Unspecified antibiotic |  |
| E931.x | Other anti-infectives ^c^ |  |
| E932.x | Hormones and synthetic substitutes |  |
| E933.x | Primarily systemic agents |  |
| E934.x | Agents primarily affecting blood constituents |  |
| E935.x | Analgesics, antipyretics, and antirheumatics |  |
| E936.x | Anticonvulsants and anti-Parkinsonism agents ^d^ |  |
| E937.x | Sedatives and hypnotics |  |
| E938.x | Other central nervous system depressants and anesthetics |  |
| E939.x | Psychotropic agents |  |
| E940.x | Central nervous system stimulants |  |
| E941.x | Drugs primarily affecting the autonomic nervous system |  |
| E942.x | Agents primarily affecting the cardiovascular system |  |
| E943.x | Agents primarily affecting the gastrointestinal system |  |
| E944.x | Water, mineral, and uric acid metabolism drugs ^e^ |  |
| E945.x | Agents primarily acting on the smooth and skeletal muscles and respiratory system |  |
| E946.x | Agents primarily affecting skin and mucous membrane, ophthalmological, otorhinolaryngological, and dental drugs |  |
| E947.x | Other and unspecified drugs and medicinal substances |  |
| E948.x | Bacterial vaccines |  |
| E949.x | Other vaccines and biological substances |  |
| C. ICD-9-CM diagnosis codes used to identify potentially related comorbidities | |  |
| ICD-9-CM codes | **Description** |  |
| 585.x | Chronic kidney disease |  |
| 401.1 ^f^ | Essential hypertension, benign |  |
| 401.9 ^f^ | Essential hypertension, unspecified |  |
| 402.1 ^f^ | Hypertensive heart disease, benign |  |
| 402.9 ^f^ | Hypertensive heart disease, unspecified |  |
| 404.1 ^f^ | Hypertensive heart and chronic disease, benign |  |
| 404.9 ^f^ | Hypertensive heart and chronic disease, unspecified |  |
| 405.1 ^f^ | Secondary hypertension, benign |  |
| 405.9 ^f^ | Secondary hypertension, unspecified |  |
| 428.x | Heart failure |  |
| 250.x | Diabetes mellitus |  |
| 042 ^g^ | Human immunodeficiency virus (HIV) disease |  |
| V08 ^g^ | Asymptomatic HIV infection status |  |
| 070.2 ^h^ | Viral hepatitis B with hepatic coma |  |
| 070.3 ^h^ | Viral hepatitis B without mention of hepatic coma |  |
| 070.44 ^h^ | Chronic hepatitis C with hepatic coma |  |
| 070.54 ^h^ | Chronic hepatitis C without mention of hepatic coma |  |
| 070.6 ^h^ | Unspecified viral hepatitis with hepatic coma |  |
| 070.9 ^h^ | Unspecified viral hepatitis without mention of hepatic coma |  |
| 570 ^h^ | Acute and subacute necrosis of liver |  |
| 571.x ^h^ | Chronic liver disease and cirrhosis |  |
| 572.2 ^h^ | Hepatic coma |  |
| 572.3 ^h^ | Portal hypertension |  |
| 572.4 ^h^ | Hepatorenal syndrome |  |
| 572.8 ^h^ | Other sequelae of chronic liver disease |  |
| 573.3 ^h^ | Hepatitis, unspecified |  |
| 573.4 ^h^ | Hepatic infarction |  |
| 573.8 ^h^ | Other specified disorders of liver |  |
| 573.9 ^h^ | Unspecified disorder of liver |  |
| V42.7 ^h^ | Liver replaced by transplant |  |

^a^ These ICD-9-CM codes were used to identify episodes with associated diagnosis of erythema multiforme.

^b^ Encompasses: E930.1 - Antifungal antibiotics; E930.2 - Chloramphenicol group; E930.3 - Erythromycin and other macrolides; E930.4 – Tetracycline group; E930.5 – Cephalosporin group; E930.7 – Antineoplastic antibiotics; and E930.8 – Other specified antibiotics.

^c^ We separately assessed Sulfonamides (E931.0); Antiviral drugs (E931.7); and Antimycobacterial drugs (E931.8 and E930.6).

^d^ We separately assessed Anticonvulsants (E936.0-E936.3)

^e^ We separately assessed Uric acid metabolism drugs (E944.7)

^f^ These ICD-9-CM codes were used to identify episodes with associated diagnosis of hypertension.

^f^ These ICD-9-CM codes were used to identify episodes with associated diagnosis of HIV infection.

h These ICD-9-CM codes were used to identify episodes with associated diagnosis of liver disease.

**Legend:** ICD-9-CM codes selected for identification of hospitalizations with associate diagnosis of drug-related Stevens-Johnson syndrome/toxic epidermal necrolysis or erythema multiforme, and possibly related comorbidities.
