# Supplementary material for: Stevens–Johnson syndrome/toxic epidermal necrolysis and erythema multiforme drug-related hospitalisations in a national administrative database
Source: Clin Transl Allergy. 2018 Jan 22;8:2. doi: 10.1186/s13601-017-0188-1 (PMC5776772; doi:10.1186/s13601-017-0188-1)
Supplement: Supplementary file 2 — Additional file 2: Table 2. Annual incidence of hospitalisations with associated diagnosis of erythema multiforme (EM) or Stevens-Johnson syndrome/toxic epidermal necrolysis(SJS/TEN). [file 13601_2017_188_MOESM2_ESM.docx]

**Additional table 2.** Annual incidence of hospitalizations with associated diagnosis of erythema multiforme (EM) or Stevens-Johnson syndrome/toxic epidermal necrolysis(SJS/TEN).

| Year | Cutaneous adverse reactions – *n* (annual incidence *per* million inhabitants) | |  | SJS/TEN – *n* (annual incidence *per* million inhabitants) | | |
| --- | --- | --- | --- | --- | --- | --- |
|  | **EM** | **SJS/TEN** |  | **Stevens-Johnson Syndrome** | **SJS-TEN overlap^a^** | **Toxic Epidermal Necrolysis** |
| 2009 | 32 (3.2) | 7 (0.7) |  | 5 (0.5) | 1 (0.1) | 1 (0.1) |
| 2010 | 16 (1.6) | 11 (1.1) |  | 6 (0.6) | 0 | 5 (0.5) |
| 2011 | 19 (1.9) | 21 (2.1) |  | 13 (1.3) | 0 | 8 (0.8) |
| 2012 | 17 (1.7) | 29 (2.9) |  | 14 (1.4) | 5 (0.5) | 10 (1.0) |
| 2013 | 17 (1.7) | 27 (2.7) |  | 17 (1.7) | 6 (0.6) | 4 (0.4) |
| 2014 | 21 (2.1) | 37 (3.7) |  | 18 (1.8) | 6 (0.6) | 13 (1.3) |
| Total | 122 (12.2) | 132 (13.2) |  | 73 (7.3) | 18 (1.8) | 41 (4.1) |

^a^ Stevens-Johnson and toxic epidermal necrolysis overlap syndrome.
